# Establishment and lineage dynamics of the SARS-CoV-2 epidemic in the UK

**DOI:** 10.1126/science.abf2946

**Published:** 2021-01-08

**Authors:** Louis du Plessis, John T. McCrone, Alexander E. Zarebski, Verity Hill, Christopher Ruis, Bernardo Gutierrez, Jayna Raghwani, Jordan Ashworth, Rachel Colquhoun, Thomas R. Connor, Nuno R. Faria, Ben Jackson, Nicholas J. Loman, Áine O’Toole, Samuel M. Nicholls, Kris V. Parag, Emily Scher, Tetyana I. Vasylyeva, Erik M. Volz, Alexander Watts, Isaac I. Bogoch, Kamran Khan, COVID-19 Genomics UK (COG-UK) Consortium, David M. Aanensen, Moritz U. G. Kraemer, Andrew Rambaut, Oliver G. Pybus

**Affiliations:** 1 Department of Zoology, University of Oxford, Oxford, UK.; 2 Institute of Evolutionary Biology, University of Edinburgh, Edinburgh, UK.; 3 Molecular Immunity Unit, Department of Medicine, University of Cambridge, Cambridge, UK.; 4 Department of Veterinary Medicine, University of Cambridge, Cambridge, UK.; 5 School of Biological and Environmental Sciences, Universidad San Francisco de Quito USFQ, Quito, Ecuador.; 6 School of Biosciences, Cardiff University, Cardiff, UK.; 7 Pathogen Genomics Unit, Public Health Wales NHS Trust, Cardiff, UK.; 8 MRC Centre for Global Infectious Disease Analysis, J-IDEA, Imperial College London, London, UK.; 9 Institute of Microbiology and Infection, University of Birmingham, Birmingham, UK.; 10 Department of Medicine, University of Toronto, Toronto, Canada.; 11 Divisions of General Internal Medicine and Infectious Diseases, University Health Network, Toronto, Canada.; 12 Li Ka Shing Knowledge Institute, St. Michael’s Hospital, Toronto, Canada.; 13 BlueDot, Toronto, Canada.; 14 Centre for Genomic Pathogen Surveillance, Wellcome Genome Campus, Hinxton, UK.; 15 Big Data Institute, Li Ka Shing Centre for Health Information and Discovery, Nuffield Department of Medicine, University of Oxford, Oxford, UK.; 16 Department of Pathobiology and Population Sciences, Royal Veterinary College London, London, UK.

## Abstract

The UK’s COVID-19 epidemic during early 2020 was one of world’s largest and unusually well represented by virus genomic sampling. Here we reveal the fine-scale genetic lineage structure of this epidemic through analysis of 50,887 SARS-CoV-2 genomes, including 26,181 from the UK sampled throughout the country’s first wave of infection. Using large-scale phylogenetic analyses, combined with epidemiological and travel data, we quantify the size, spatio-temporal origins and persistence of genetically-distinct UK transmission lineages. Rapid fluctuations in virus importation rates resulted in >1000 lineages; those introduced prior to national lockdown tended to be larger and more dispersed. Lineage importation and regional lineage diversity declined after lockdown, while lineage elimination was size-dependent. We discuss the implications of our genetic perspective on transmission dynamics for COVID-19 epidemiology and control.

Infectious disease epidemics are composed of chains of transmission, yet surprisingly little is known about how co-circulating transmission lineages vary in size, spatial distribution, and persistence, and how key properties such as epidemic size and duration arise from their combined action. While individual-level contact tracing investigations can reconstruct the structure of small-scale transmission clusters [e.g., (*1*–*3*)] they cannot be extended practically to large national epidemics. However, recent studies of Ebola, Zika, influenza and other viruses have demonstrated that virus emergence and spread can be instead tracked using large-scale pathogen genome sequencing [e.g., (*4*–*7*)]. Such studies show that regional epidemics can be highly dynamic at the genetic level, with recurrent importation and extinction of transmission chains within a given location. In addition to measuring genetic diversity, understanding pathogen lineage dynamics can help target interventions effectively [e.g., (*8*, *9*)], track variants with potentially different phenotypes [e.g., (*10*, *11*)], and improve the interpretation of incidence data [e.g., (*12*, *13*)].

The rate and scale of virus genome sequencing worldwide during the COVID-19 pandemic has been unprecedented, with >100,000 SARS-CoV-2 genomes shared online by 1 October 2020 (*14*). Notably, approximately half of these represent UK infections and were generated by the national COVID-19 Genomics UK (COG-UK) consortium (*15*). The UK experienced one of the largest epidemics worldwide during the first half of 2020. Numbers of positive SARS-CoV-2 tests rose in March and peaked in April; by 26 June there had been 40,453 nationally-notified COVID-19 deaths in the UK (deaths occurring ≤28 days of first positive test; (*16*). Here, we combine this large genomic data set with epidemiological and travel data to provide a full characterisation of the genetic structure and lineage dynamics of the UK epidemic.

Our study encompasses the initial epidemic wave of COVID-19 in the UK and comprises all SARS-CoV-2 genomes available before 26 June 2020 (50,887 genomes, of which 26,181 were from the UK; Fig. 1A) (*17*). The data represents genomes from 9.29% of confirmed UK COVID-19 cases by 26 June (*16*). Further, using an estimate of the actual size of the UK epidemic (*18*) we infer virus genomes were generated for 0.66% (95% CI = 0.46-0.95%) of all UK infections by 5^th^ May (Fig. 1B).

**Fig. 1:**
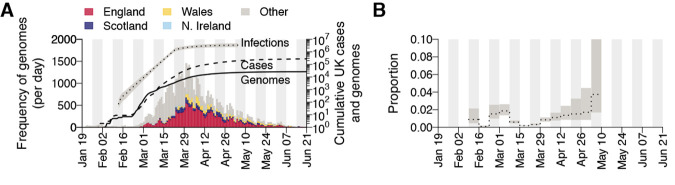
Genomic sequence data. (**A**) Collection dates of the 50,887 genomes analyzed here (left-hand axis). Genomes are colored by sampling location (England = red, Scotland = dark blue, Wales = yellow, Northern Ireland = light blue, elsewhere = grey). The solid line shows the cumulative number of UK virus genomes (right-hand axis). The dashed and dotted lines show, respectively, the cumulative number of laboratory-confirmed UK cases (by specimen date) and the estimated number of UK infections (*18*); grey shading = 95% CI; right-hand axis). Due to retrospective screening, the cumulative number of genomes early in the epidemic exceeds that of confirmed cases. (**B**) Proportion of weekly estimated UK infections (*18*) included in our genome sequence dataset.

## Genetic structure and lineage dynamics of the UK epidemic from January to June

**Fig. 2:**
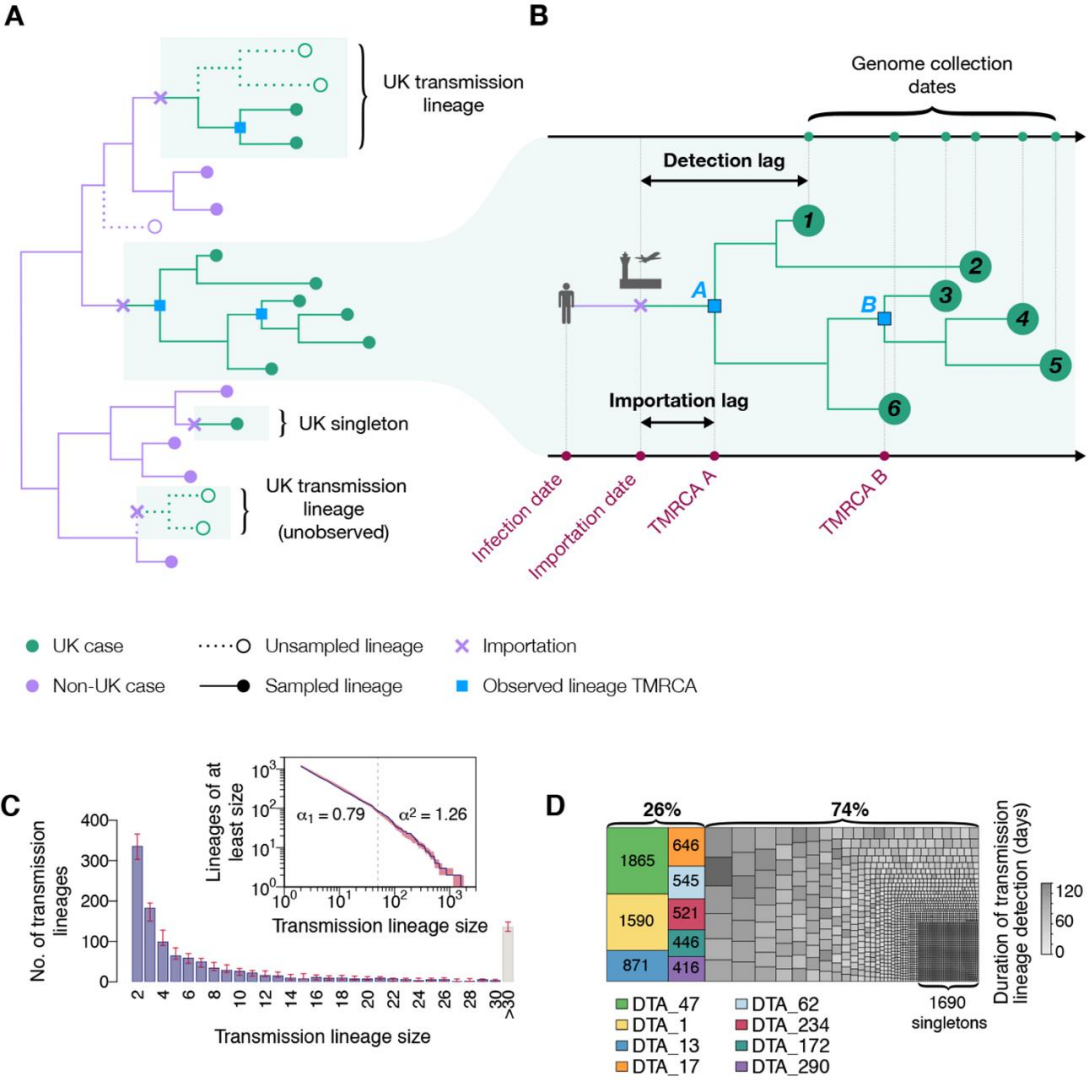
Structure of UK transmission lineages detected through genome sampling. (**A**) Figurative illustration of the international context of UK transmission lineages. Note only half of the cases in the top UK transmission lineage are observed and the bottom UK transmission lineage is unobserved. To be detected, a UK transmission lineage must contain two or more sampled genomes; singletons are not classified here as UK transmission lineages. (**B**) Detailed view of one of the UK transmission lineages from (A), used to illustrate the terms TMRCA, detection lag, and importation lag. The lineage TMRCA is sample-dependent; for example, TMRCA A is observed if genomes 1–6 are sampled and TMRCA B is observed if only genomes 3–5 are sampled. (**C**) Distribution of UK transmission lineage sizes. Blue bars show the number of transmission lineages of each size (red bars = 95% HPD of these sizes across the posterior tree distribution). Inset: the corresponding cumulative frequency distribution of lineage size (blue line), on double logarithmic axes (red shading = 95% HPD of this distribution across the posterior tree distribution). Values either side of vertical dashed line show coefficients of power-law distributions (*P*[*X* ≥ *x*] ~ *x*^–α^) fitted to lineages containing ≤50 (α_1_) and >50 (α_2_) virus genomes, respectively. (**D**) Partition of 26,181 UK genomes into UK transmission lineages and singletons, colored by (i) lineage, for the 8 largest lineages, or (ii) duration of lineage detection (time between the lineage’s oldest and most recent genomes) for the remainder. The sizes of the 8 largest lineages are also shown in the figure.

We first sought to identify and enumerate all independently introduced, genetically-distinct chains of infection within the UK. We developed a large-scale molecular clock phylogenetic pipeline to identify “UK transmission lineages” that (i) contain two or more UK genomes and (ii) descend from an ancestral lineage inferred to exist outside of the UK (Fig. 2, A and B). Sources of statistical uncertainty in lineage assignation were taken into account (*17*). We identified a total of 1179 (95% HPD 1143-1286) UK transmission lineages. Although each is intended to capture a chain of local transmission arising from a single importation event, some UK transmission lineages will be unobserved or aggregated due to limited SARS-CoV-2 genetic diversity (*19*) or incomplete or uneven genome sampling (*20*, *21*). Therefore we expect this number to be an underestimate (*17*). In our phylogenetic analysis 1650 (95% HPD 1611-1783) UK genomes could not be allocated to a UK transmission lineage (singletons). Had more genomes been sequenced, it is likely that many of these singletons would have been assigned to a UK transmission lineage. Further, many singleton importations are likely to be unobserved.

**Fig. 3:**
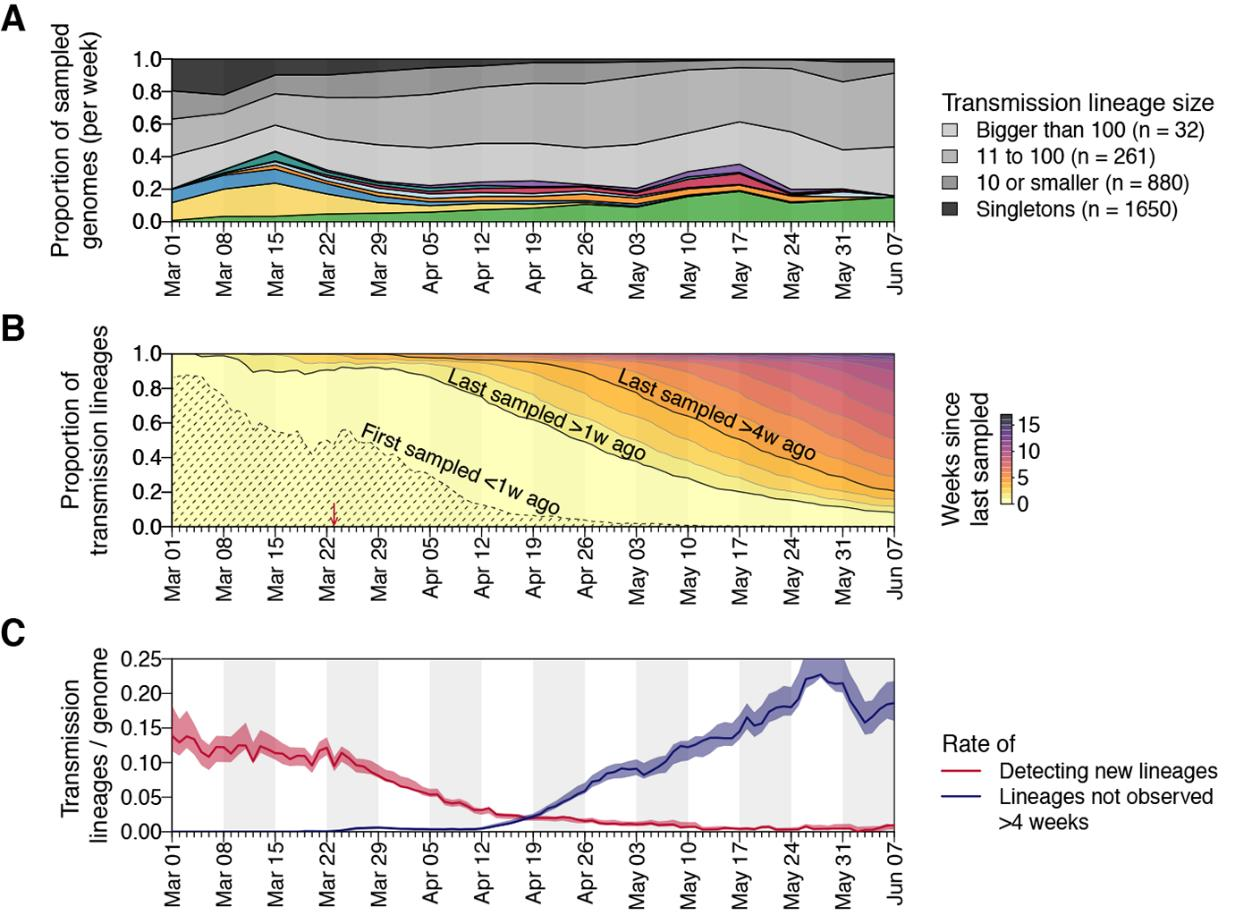
Dynamics of UK transmission lineages. (**A**) Lineage size breakdown of UK genomes collected each week. Colors of the 8 largest lineages are as depicted in Fig. 2D. (**B**) Trends through time in the detection of UK transmission lineages. For each day, all lineages detected up to that day are colored by the time since the transmission lineage was last sampled. Isoclines correspond to weeks. Shaded area = transmission lineages that were first sampled <1 week ago. The red arrow indicates the start of the UK lockdown. (**C**) Red line = daily rate of detecting new transmission lineages. Blue line = rate at which lineages have not been observed for >4 weeks, shading = 95% HPD across the posterior distribution of trees.

Most transmission lineages are small and 72.4% (95% HPD 69.3-72.9%) contain <10 genomes (Fig. 2C). However the lineage size distribution is strongly skewed and follows a power-law distribution (Fig. 2C, inset), such that the 8 largest UK transmission lineages contain >25% of all sampled UK genomes (Fig. 2D; figs. S2 to S5 show further visualizations). Although the two largest transmission lineages are estimated to comprise >1500 UK genomes each, there is phylogenetic uncertainty in their sizes (95% HPDs = 1280-2133 and 1342-2011 genomes). Since our dataset comprises only a small fraction of all UK infections, these observed lineage sizes will underestimate true lineage size. However, the true distribution of relative lineage sizes will closely match our observation, and its power-law shape indicates that almost all unobserved lineages will be small. All 8 largest lineages were first detected before the UK national lockdown was announced on 23 March and, as expected, larger lineages were observed for longer (Pearson’s r = 0.82; 95% CI = 0.8-0.83; fig. S7). The sampling frequency of lineages of varying sizes differed over time (Fig. 3A and figs. S8 and S9); while UK transmission lineages containing >100 genomes consistently accounted for >40% of weekly sampled genomes, the proportion of small transmission lineages (≤10 genomes) and singletons decreased over the course of the epidemic (Fig. 3A).

The detection of UK transmission lineages in our data changed markedly through time. In early March the epidemic was characterised by lineages first observed within the previous week (Fig. 3B). The per-genome rate of appearance of new lineages was initially high, then declined throughout March and April (Fig. 3C), such that by 1^st^ May 96.2% of sampled genomes belonged to transmission lineages that were first observed >7 days previously. By 1^st^ June, a growing number of lineages (>73%) had not been detected by genomic sampling for >4 weeks, suggesting that they were rare or had gone extinct, a result that is robust to the sampling rate (Fig. 1, A and B, and Fig. 3C). Together, these results indicate that the UK’s first epidemic wave resulted from the concurrent growth of many hundreds of independently-introduced transmission lineages, and that the introduction of non-pharmaceutical interventions (NPIs) was followed by the apparent extinction of lineages in a size-dependent manner.

## Transmission lineage diversity and geographic range

**Fig. 4:**
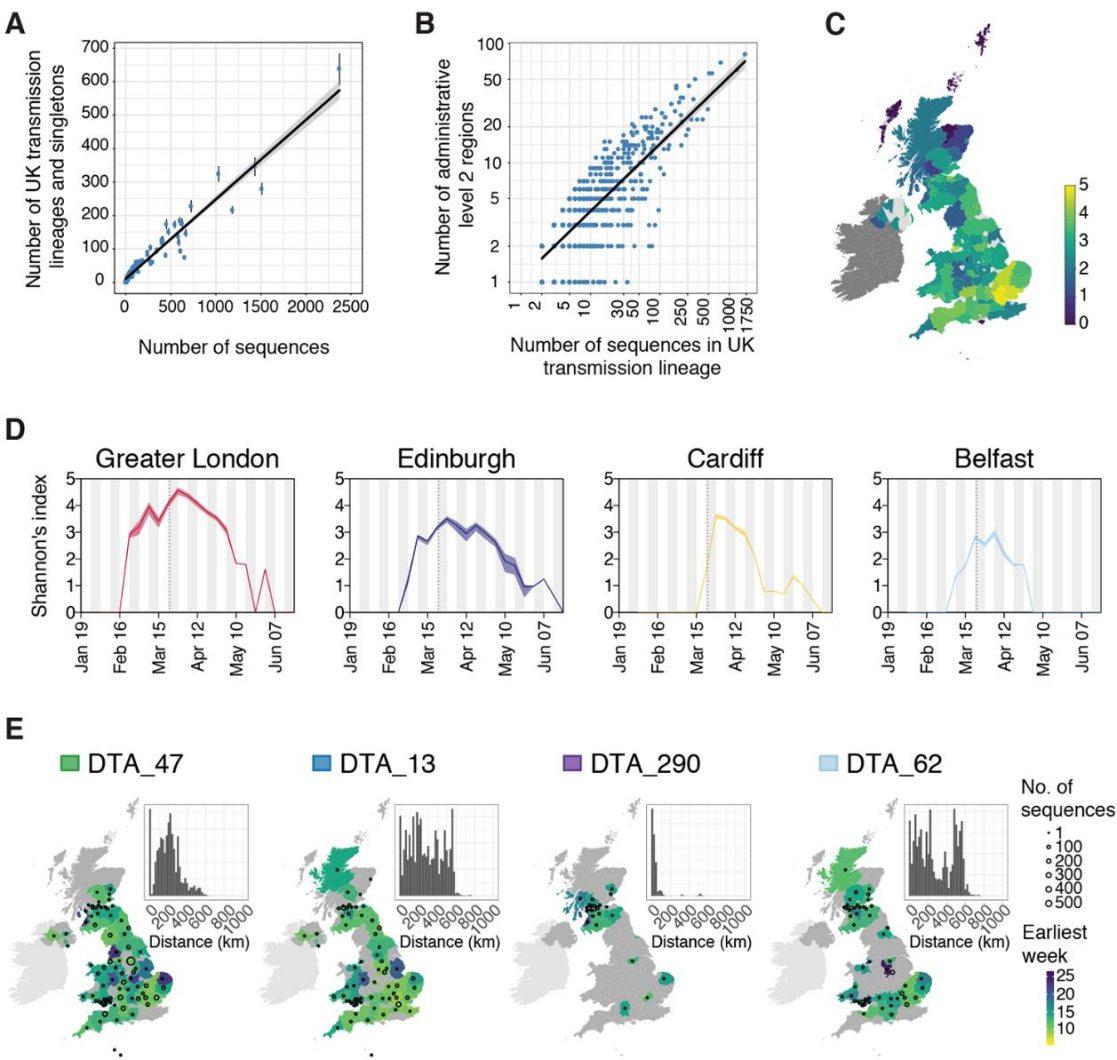
Spatial distribution of UK transmission lineages. (**A**) Correlation between the number of transmission lineages detected in each region (points = median values, bars = 95% HPD intervals) and the number of UK virus genomes from each region (Pearson’s r = 0.96, 95% CI = 0.95-0.98). (**B**) Correlation between the spatial range of each transmission lineage and the number of virus genomes it contains (Pearson’s r = 0.8, 95% CI = 0.78-0.82,) (**C**) Map showing Shannon’s index (SI) for each region, calculated across the study period (2^nd^ Feb-26^th^ Jun). Yellow colors indicate higher SI values and darker colors lower values. (**D**) SI through time for the UK national capital cities. The dotted lines indicate the start of the UK national lockdown. (**E**) Illustration of the diverse spatial range distributions of UK transmission lineages. Colors represent the week of the first detected genome in the transmission lineage in each location. Circles show the number of sampled genomes per location. Insets show the distribution of geographic distances for all sequence pairs within the lineage (see data S4 and fig. S12 for further details). Colored boxes next to lineage names are as depicted in Fig. 2D.

We also characterised the spatial distribution of UK transmission lineages using available data on 107 virus genome sampling locations, which correspond broadly to UK counties or metropolitan regions (data S1). Although genomes were not collected randomly (some lineages and regions will be over-represented due to targeted investigation of local outbreaks; e.g., (*22*) the number of UK lineages detected in each region correlates with the number of genomes sequenced (Fig. 4A, Pearson’s r = 0.96, 95% CI = 0.95-0.98) and the number of reported cases (fig. S10, Pearson’s r = 0.53, 95% CI = 0.35-0.67, data S2) in each region. Further, larger lineages were observed in more locations; every 100 additional genomes in a lineage increases its observed range by 6-7 regions (Fig. 4B; Pearson’s r = 0.8, 95% CI = 0.78-0.82). Thus, bigger regional epidemics comprised a greater diversity of transmission lineages, and larger lineages were more geographically widespread. These observations indicate substantial dissemination of a subset of lineages across the UK and suggest many regions experienced a series of introductions of new lineages from elsewhere, potentially hindering the impact of local interventions.

We quantified the substantial variation among regions in the diversity of transmission lineages present using Shannon’s index (SI; this value increases as both the number of lineages and the evenness of their frequencies increase; Fig. 4C and data S3). We observed the highest SIs in Hertfordshire (4.77), Greater London (4.62) and Essex (4.49); these locations are characterised by frequent commuter travel to/within London and proximity to major international airports (*23*). Locations with the three lowest nonzero SIs were in Scotland (Stirling = 0.96, Aberdeenshire = 1.04, Inverclyde = 1.32; Fig. 4C). We speculate that regional differences in transmission lineage diversity may be related to the level of connectedness to other regions.

To illustrate temporal trends in transmission lineage diversity, we plot SI through time for each of the UK’s national capital cities (Fig. 4D). Lineage diversities in each peaked in late March and declined after the UK national lockdown, congruent with Fig. 3, C and D. Greater London’s epidemic was the most diverse and characterised by an early, rapid rise in SI (Fig. 4D), consistent with epidemiological trends there (*16*, *24*). Belfast’s lineage diversity was notably lower (data S4 shows other locations).

We observe variation in the spatial range of individual UK transmission lineages. Although some lineages are widespread, most are more localized and the range size distribution is right-skewed (fig. S11), congruent with an observed abundance of small lineages (Figs. 2C and 4B) and biogeographic theory [e.g., (*25*)]. For example, lineage DTA_13 is geographically dispersed (>50% of sequence pairs sampled >234km apart) whereas DTA_290 is strongly local (95% of sequence pairs sampled <100km apart) and DTA_62 has multiple foci of sampled genomes (Fig. 4E and fig. S12). The national distribution of cases therefore arose from the aggregation of multiple heterogeneous lineage-specific patterns.

## Dynamics of international introduction of transmission lineages

**Fig. 5:**
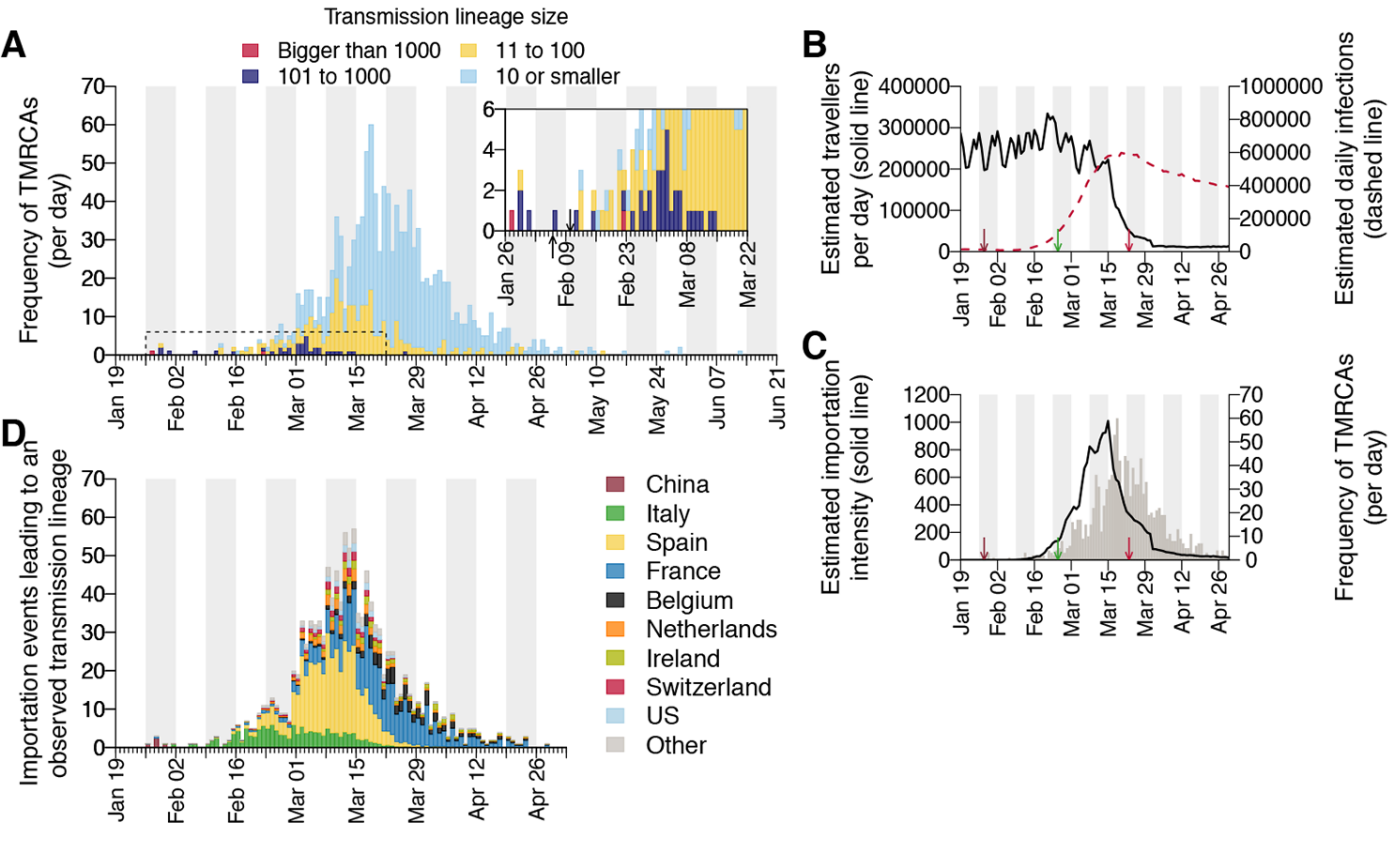
Dynamics of UK transmission lineage importation. (**A**) Histogram of lineage TMRCAs, colored by lineage size. Inset: expanded view of the days prior to UK lockdown. Left-hand arrow = collection date of the UK’s first laboratory-confirmed case; right-hand arrow = collection date of the earliest UK virus genome in our dataset. (**B**) Estimated number of inbound travellers to the UK per day (black) and estimated number of infectious cases worldwide (dashed red). Arrows here show, from left to right, dates of the first self-isolation advice for returning travellers from China, Italy, and of the start of the UK national lockdown. (**C**) Estimated importation intensity (EII) curve (black) and the histogram of lineage TMRCAs (grey). (**D**) Estimated histogram of virus lineage importation events per day, obtained from our lag model. Colors show the proportion attributable each day to inbound travel from various countries (see table S4 and figs. S19 and S20). This assignment is statistical, i.e., we cannot ascribe a specific source location to any given lineage.

The process by which transmission lineages are introduced to an area is an important aspect of early epidemic growth [e.g., (*26*)]. To investigate this at a national scale we estimated the rate and source of SARS-CoV-2 importations into the UK. Since standard phylogeographic approaches were precluded by strong biases in genome sampling among countries (*20*), we developed a new approach that combines virus phylogenetics with epidemiological and travel data. First, we estimated the TMRCA (time of the most recent common ancestor) of each UK transmission lineage (*17*). The TMRCAs of most UK lineages are dated to March and early April (median = 21^st^ March; IQR = 14^th^-29^th^ March). UK lineages with earlier TMRCAs tend to be larger and longer-lived than those whose TMRCAs postdate the national lockdown (Fig. 5A and fig. S15).

Due to incomplete sampling, TMRCAs best represent the date of the first inferred transmission event in a lineage, not its importation date (Fig. 2B). To infer the latter, and quantify the delay between importation and onward within-UK transmission, we generated daily estimates of the number of travellers arriving in the UK and of global SARS-CoV-2 infections (*17*) worldwide. Before March, the UK received ~1.75m inbound travellers per week (school holidays explain the end-February ~10% increase; Fig. 5B). International arrivals fell by ~95% during March and this reduction was maintained through April. Elsewhere, estimated numbers of infectious cases peaked in late March (Fig. 5B). We combined these two trends to generate an estimated importation intensity (EII) - a daily empirical measure of the intensity of SARS-CoV-2 importation into the UK (*17*). Since both travel volumes and epidemic incidence fluctuate rapidly over orders of magnitude, the EII is robust to other sources of variation in the relative importation risk among countries (*17*). The EII peaks in mid-March, when high UK inbound travel volumes coincided with growing numbers of infectious cases elsewhere (Fig. 5, B and C).

Crucially, the EII’s temporal profile closely matches, but precedes, that of the TMRCAs of UK transmission lineages (Fig. 5, A and C). The difference between the two represents the “importation lag”, the time elapsed between lineage importation and the first detected local transmission event (Fig. 2B). Using a statistical model (*17*), we estimate importation lag to be on average 8.22 ± 5.21 days (IQR = 3.35-15.18) across all transmission lineages. Further, importation lag is strongly size-dependent; average lag is ~10 days for lineages comprising ≤10 genomes and <1 day for lineages of >100 genomes (table S2). This size-dependency likely arises because the earliest transmission event in a lineage is more likely to be captured if it contains many genomes (Fig. 2B) (*17*). We use this model to impute an importation date for each UK transmission lineage (Fig. 5D). Importation was unexpectedly dynamic, rising and falling substantially over only 4 weeks, hence 80% of importations (that gave rise to detectable UK transmission lineages) occurred between 27 February and 30 March. The delay between the inferred date of importation and the first genomic detection of each lineage was 14.13 ± 5.61 days on average (IQR = 10-18) and declined through time (tables S2 and S3).

To investigate country-specific contributions to virus importation we generated separate importation intensity (EII) curves for each country (fig. S17). Using these values, we estimated the numbers of inferred importations each day attributable to inbound travel from each source location. This assignment is statistical and does not take the effects of superspreading events into account. As with the rate of importation (Fig. 5A), the relative contributions of arrivals from different countries were dynamic (Fig. 5D). Dominant source locations shifted rapidly in February and March and the diversity of source locations increased in mid-March (fig. S17). Earliest importations were most likely from China or elsewhere in Asia but were rare compared to those from Europe. Over our study period we infer ~33% of UK transmission lineages stemmed from arrivals from Spain, 29% from France, 12% from Italy and 26% from elsewhere (fig. S20 and table S4). These large-scale trends were not apparent from individual-level travel histories; routine collection of such data ceased on 12 March (*27*).

## Conclusions

The exceptional size of our genomic survey provides insight into the micro-epidemiological patterns that underlie the features of a large, national COVID-19 epidemic, allowing us to quantify the abundance, size distribution, and spatial range of transmission lineages. Pre-lockdown, high travel volumes and few restrictions on international arrivals (Fig. 5B and table S5) led to the establishment and co-circulation of >1000 identifiable UK transmission lineages (Fig. 5A), jointly contributing to accelerated epidemic growth that quickly exceeded national contact tracing capacity (*27*). The relative contributions of importation and local transmission to initial epidemic dynamics under such circumstances warrants further investigation. We expect similar trends occurred in other countries with comparably large epidemics and high international travel volumes; virus genomic studies from regions with smaller or controlled COVID-19 epidemics have reported high importation rates followed by more transient lineage persistence [e.g., (*28*–*30*)].

Earlier lineages were larger, more dispersed, and harder to eliminate, highlighting the importance of rapid or pre-emptive interventions in reducing transmission [e.g., (*31*–*33*)]. The high heterogeneity in SARS-CoV-2 transmission at the individual level (*34*–*36*) appears to extend to whole transmission lineages, such that >75% of sampled viruses belong to the top 20% of lineages ranked by size. While the national lockdown coincided with limited importation and reduced regional lineage diversity, its impact on lineage extinction was size-dependent (Fig. 3, B and C). The over-dispersed nature of SARS-CoV-2 transmission likely exacerbated this effect (*37*), thereby favoring, as *R_t_* declined, greater survival of larger widespread lineages and faster local elimination of lineages in low prevalence regions. The degree to which the surviving lineages contributed to the UK’s ongoing second epidemic, including the effect of specific mutations on lineage growth rates [e.g., (*11*)], is currently under investigation. The transmission structure and dynamics measured here provide a new context in which future public health actions at regional, national, and international scales should be planned and evaluated.

## Supplementary Materials

science.sciencemag.org/cgi/content/full/science.abf2946/DC1 Materials and Methods Figs. S1 to S20 Tables S1 to S5 Data S1 to S5 References (*39**–**68*)
